# Transcriptional Profile of Kidney from Type 2 Diabetic db/db Mice

**DOI:** 10.1155/2017/8391253

**Published:** 2017-01-23

**Authors:** Haojun Zhang, Tingting Zhao, Zhiguo Li, Meihua Yan, Hailing Zhao, Bin Zhu, Ping Li

**Affiliations:** ^1^Beijing Key Lab Immune-Mediated Inflammatory Diseases, Institute of Clinical Medical Sciences, China-Japan Friendship Hospital, Beijing, China; ^2^Department of Medical Research Center, International Science and Technology Cooperation Base of Geriatric Medicine, North China University of Science and Technology, Tangshan, China

## Abstract

Diabetic nephropathy (DN), a common diabetic microvascular complication, is characterized by progressive glomerular sclerosis and tubulointerstitial fibrosis. However, the underlying mechanisms involved in DN remain to be elucidated. We explored changes in the transcriptional profile in spontaneous type 2 diabetic db/db mice by using the cDNA microarray. Compared with control db/m mice, the db/db mice exhibited marked increases in body weight, kidney weight, and urinary albumin excretion. Renal histological analysis revealed mesangial expansion and thickness of the basement membrane in the kidney of the db/db mice. A total of 355 differentially expressed genes (DEGs) were identified by microarray analysis. Pathway enrichment analysis suggested that biological oxidation, bile acid metabolism, and steroid hormone synthesis were the 3 major significant pathways. The top 10 hub genes were selected from the constructed PPI network of DEGs, including* Ccnb2* and* Nr1i2*, which remained largely unclear in DN. We believe that our study can help elucidate the molecular mechanisms underlying DN.

## 1. Introduction

With the increasing prevalence of diabetes worldwide, diabetic nephropathy, a serious and major microvascular complication of diabetes mellitus (DM), has also become a global problem affecting about 40% of patients with diabetes and is the primary cause of end-stage renal diseases (ESRD) in developed countries [1, 2]. DN is characterized by excessive accumulation of extracellular matrix (ECM) with thickening of glomerular and tubular basement membranes and increased mesangial materials [3]. Therapies for DN remain clinically limited thus far [4]. Although attempts including intensive control of hyperglycemia and hypertension reduced albuminuria accompanied with delay in DN progression, many patients still experience exacerbated renal injuries and develop ESRD [2]. A better understanding of the molecular mechanisms of DN can thus help establish effective therapeutic strategies for the condition.

To fundamentally understand and elucidate the mechanism of DN, high-throughput microarrays could be used to investigate the underlying genetic characteristic of processes involved in DN. These techniques have been widely used in screening the potential targets for DN. Distinct patterns of molecules and pathways between glomeruli and tubulointerstitial compartment were identified by transcriptome analysis of human DN [5]. The transcriptional profile from renal biopsies revealed activation of the NF-kB pathway in DN [6]. Watanabe et al. indicated that the macrophage migration inhibitory factor could be an important cytokine for induction of microalbuminuria by the cDNA microarray [7]. Thus, microarray analysis can effectively identify key molecular events and pathways involved in DN.

Animal models exhibit a remarkable advantage when difficulties arise in clinical trials. Db/db mice were considered good animal models for DN. The G-to-T point mutation of leptin receptor mutation (LepRdb/db) from db/db mice results in defective responses to leptin, leading to the development of hyperphagia, obesity, hyperlipidemia, hyperinsulinemia, insulin resistance, and diabetes. Db/db mice could develop mild kidney damage, which is similar to early-stage of human type 2 DN. Thus, db/db mice are commonly used to investigate the mechanisms of renal injury associated with type 2 diabetes [8].

In the current study, we employed global microarray analysis combined with bioinformatics to explore the differential gene expression in db/db and db/m mice in order to identify candidate genes that may be involved in the development of DN.

## 2. Materials and Methods

### 2.1. Animal Experimentation

Male db/db mice (*n* = 6) at 10 weeks old and their nondiabetic db/m littermates (*n* = 6) were purchased from Beijing Vital River Laboratory Animal Co. Ltd. (Beijing, China). Animals were given free access to standard chow and water for 12 weeks. At the end of the experiment, all mice were weighed, and individual 24 h urine collections were obtained using metabolic cages. Subsequently, all animals were sacrificed at 22 weeks of age. Their blood was collected, and their kidneys were harvested, weighed, and immediately frozen in liquid nitrogen for further analysis. The mice were housed in an animal care facility at China-Japan Friendship Hospital. The protocol was approved by the ethics committee of the Institute of Clinical Medical Sciences at China-Japan Friendship Hospital and then executed as specified in the National Institutes of Health Guide for the Care and Use of Laboratory Animals.

### 2.2. Urinary Albumin Excretion and Renal Histology

Urinary albumin concentrations were determined using Mouse Albumin ELISA Quantitation Set (Bethyl Laboratories Inc., Montgomery, TX) in accordance with the manufacturer's instructions.

Dissected kidney samples were fixed in buffered formalin (10%) for 24 h, dehydrated, embedded in paraffin, sectioned at 3 *μ*m thickness, and mounted on slides. The paraffin sections were stained with periodic acid-Schiff (PAS) and examined under light microscopy.

### 2.3. RNA Extraction, Amplification, Labeling, and Hybridization

Renal cortices were carefully isolated from 3 mice in db/db and db/m group, respectively, for microarray analysis. Total RNA were extracted using TriZol Reagent (Invitrogen, Carlsbad, CA) in accordance with the manufacturer's instructions. Removal of contaminating genomic DNA was conducted using DNase I digested for 15 min at 37°C. After being cleaned up with RNeasy Kit (Qiagen, Hilden, Germany), the RNA quantities and qualities were determined by spectrophotometry and 1% formaldehyde denaturing gel electrophoresis, respectively. Samples with bright bands of ribosomal 28S to 18S RNAs in a ratio >1.5 : 1 were used for microarray analysis.

Microarray experiments were performed by CapitalBio Corporation (Beijing, China), a service provider authorized by Affymetrix, Inc. (Santa Clara, CA) in accordance with the Affymetrix GeneChip® manual. As much as 100 ng of total RNA was used for cDNA synthesis. Biotin-tagged cRNA was produced using the GeneChip IVT Labeling Kit (Affymetrix). Subsequently, 15 *μ*g fragmented cRNA, with Control Oligo B2 and eukaryotic hybridization controls (bioB, bioC, bioD, cre), was hybridized to the Affymetrix Mouse Genome 430 2.0 Array (Affymetrix, Santa Clara, CA) at 45°C for 16 h (Affymetrix GeneChip Hybridization Oven 640). After hybridization, the GeneChip arrays were washed and then stained with streptavidin phycoerythrinonan with Affymetrix GeneChip Fluidics Station 450, followed by scanning with Affymetrix GeneChip Scanner 3000 7G.

Data were analyzed using Affymetrix Expression Console and Transcriptome Analysis Console (TAC) software. The gene array was run in triplicate, and the significance of the difference for each gene was determined by one-way ANOVA. Differentially regulated genes (DEGs) were defined as genes with a 1.5-fold and greater change over controls with *p* < 0.05. These microarray data have been submitted to the Gene Expression Omnibus repository and are accessible through accession number GSE87359.

### 2.4. Enrichment Analysis for GO and Pathway

The software ClueGO 2.0.6 for “Cytoscape 3.0.1” was used to apply the “Function” analysis mode and the “Compare” cluster analysis type (cluster 1* * = * *upregulated genes and cluster 2 * *=* * downregulated genes). The statistical test used for the enrichment was based on a two-sided hypergeometric option with the Bonferroni correction, a *p* value less than 0.05, and a kappa score of 0.40. The pathway databases included KEGG, Reactome, and Wiki Pathways. The Gene Ontology (GO) databases included the biological process and molecular function Gene Ontology, updated to 10.09.2013. The Benjamini-Hochberg false discovery rate was set to 0.05.

### 2.5. Construction of PPI Network

The PPI network of DEGs in each group was constructed in the Search Tool for the Retrieval of Interacting Genes (STRING) database using Cytoscape 2.8, a free software package for visualizing, modeling, and analyzing the integration of biomolecular interaction networks with high-throughput expression data and other molecular states. The interactive pattern degree was set at 0.4.

### 2.6. Validation of DEGs by Quantitative PCR (qPCR)

The expression levels of 14 DEGs (highly upregulated or downregulated in db/db mice) were measured by qPCR in triplicate for the technical validation of microarray data. Results were expressed as fold expression relative to the expression in the control group by using the delta-delta Ct (ΔΔCt) method. The level of *β*-actin RNA was used as an internal standard. All of these primers are listed in Table 1.

### 2.7. Statistical Analysis

Data are presented as means ± SEM and compared using Student's *t*-test. *p* < 0.05 was considered statistically significant.

## 3. Results

### 3.1. Manifestation of Type 2 DN from db/db Mice

**Figure 1:**
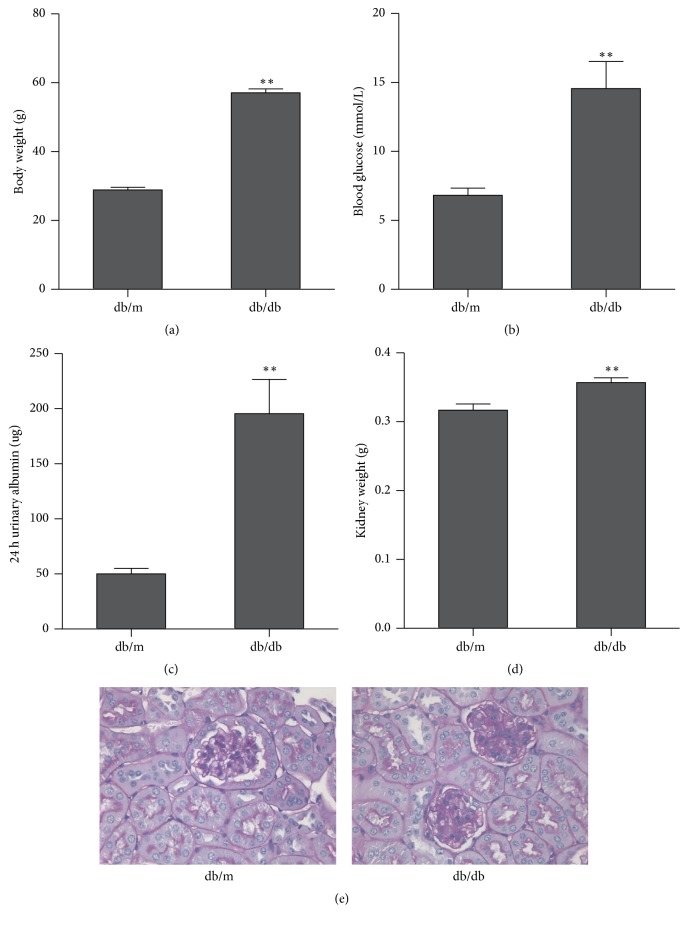
Comparison between db/db mice and db/m mice in terms of body weight (a), blood glucose (b), 24 h urinary albumin (c), and kidney weight (d). Renal histological changes are denoted by (e). Data presented as mean + SEM ^*∗∗*^*p* < 0.01, db/db versus db/m.

As shown in Figure 1, body weight and blood glucose in db/db mice were markedly higher than those in db/m mice. Compared with those of the control db/m mice, significant increases in 24 h urinary albumin and kidney weight were indicated in the db/db mice. Histological examination demonstrated a mild accumulation of mesangial matrix. Thickening of the glomerular base membrane was observed in db/db mice. No remarkable changes in the tubulointerstitium were observed in the model.

### 3.2. Identification of Differentiated Genes (DEGs)

**Figure 2:**
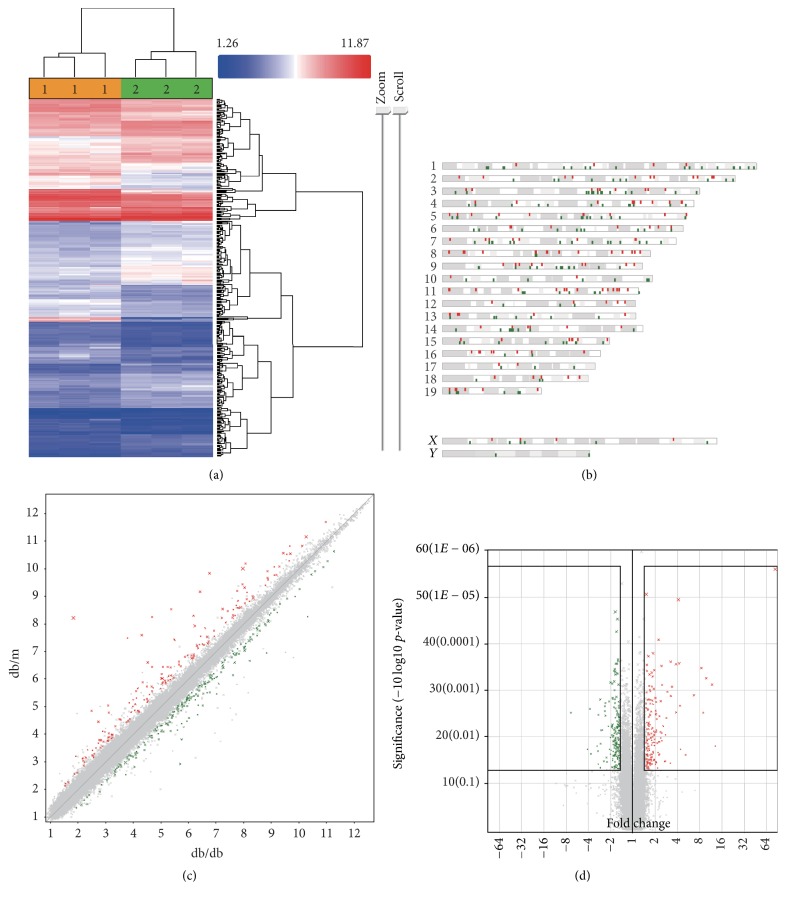
Identification of DEGs in the transcriptional profile from db/db and db/m mice at 22 w old. (a) Signal values are plotted and clustered in a heat map to determine overt differences in the signal between the 2 samples by TAC. (b) Chromosome distribution analysis shows where induced or repressed genes in db/db mice are located in the mouse genome. (c) Fold change calculated using the TAC software is exhibited as a scatter plot. Genes upregulated in db/db mice are shown in red, whereas downregulated genes are shown in green. (d) A fold change for each gene exceeding 1.5-fold change is plotted against significance as calculated by one-way ANOVA in a volcano plot. DEGs within the black boxes are considered for further ontology and pathway analysis.

In the study, a cutoff of 1.5-fold change or greater was used to selectively analyze genes that were altered by a significantly higher margin compared with normal controls. After filtering, we found that 164 genes were downregulated and 191 genes were upregulated in the db/db mice.  Figure 2(a) shows a heat map distribution of those DEGs. The genes shown in red indicate a high signal, whereas those in green indicate a low signal. The heat map demonstrated that several areas with highly altered (upregulated or downregulated) signals were shown in db/db mice compared with db/m mice. All analyses were performed using Transcriptome Analysis Console (Affymetrix). The genes were evenly distributed among the chromosomes, with no significant clustering on any one chromosome, which could suggest site-specific gene induction (Figure 2(b)). These genes are represented as a scatter plot (Figure 2(c)) showing the upregulated genes in red and the downregulated genes in green. These DEGs were plotted against significance (Figure 2(d)), with the red dots in Figure 2(c) denoting significantly induced genes and the green dots in Figure 2(c) denoting significantly repressed genes. These figures show that the gene expression profile in db/db markedly differs from that in db/m mice.

### 3.3. GO Term Enrichment Analysis of DEGs

**Figure 3:**
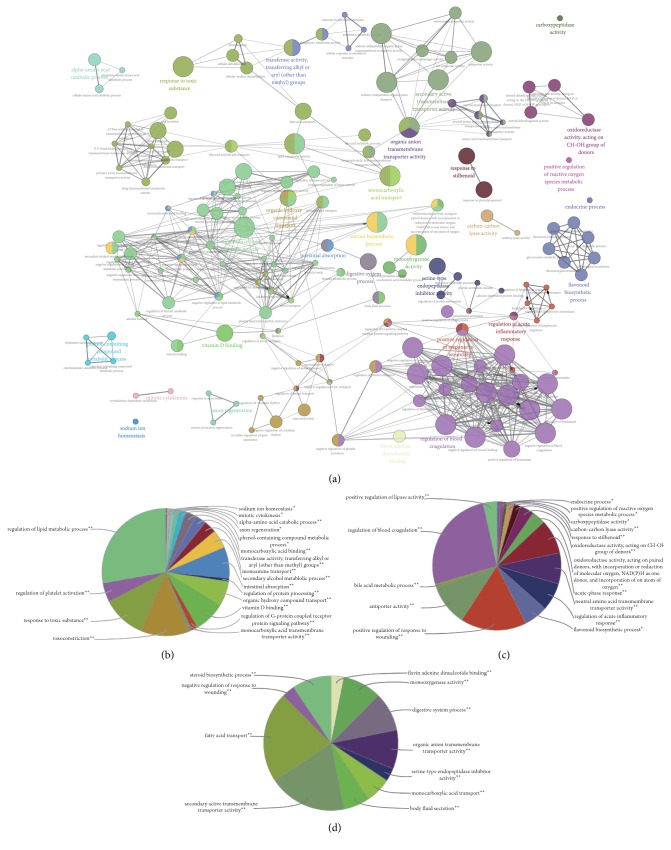
Functionally grouped network of enriched GO categories generated for the upregulated and downregulated genes. GO terms are represented as nodes. Functionally related groups partially overlap (a). Node pie charts represent the biological process analysis for upregulated genes (b) and downregulated genes (c) and uncertain genes (d).

Using the Cytoscape plug-in ClueGO, we performed Gene Ontology enrichment analysis to focus on the GO categories and thus understand the biological functions associated with DN. In general, 232 GO terms were significantly enriched, as shown in Figure 3(a). The top 10 most significant GO terms are listed in Table 2 by their *p* values. These GO terms were categorized into 29 subgroups. Specifically, the downregulated genes were mainly related to regulation of lipid metabolic process, regulation of platelet cell cycle, ATPase activity, and nucleoside-triphosphatase activity (Figure 3(b)). Meanwhile, the upregulated genes were related to kinase activity, transcription factor activity, and response to reactive oxygen species (Figure 3(c)).

### 3.4. Pathway Enrichment Analysis of DEGs

**Figure 4:**
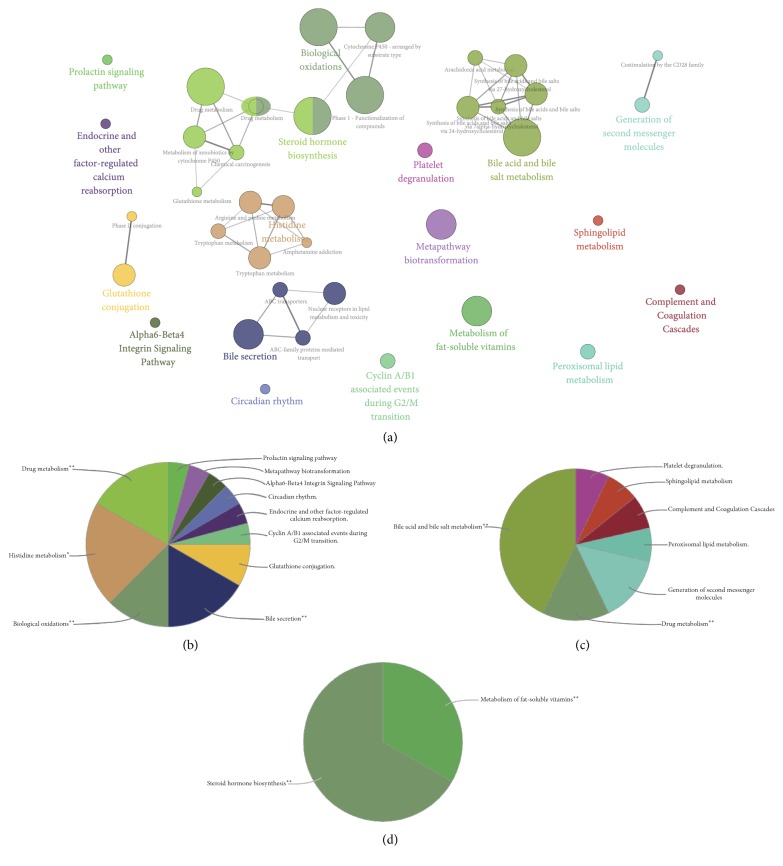
Functionally grouped network of enriched pathway categories was generated for the upregulated and downregulated genes. Pathway terms are represented as nodes. Functionally related groups partially overlap (a). The node pie charts represent pathway analyses for upregulated genes (b) and downregulated genes (c) and uncertain genes (d).

Furthermore, in the pathway enrichment analysis, 41 pathway terms were significantly enriched, as shown in Figure 4(a). The top 10 most significant pathway terms are listed in Table 3 by the *p* value. These pathway terms have been categorized into 18 subgroups. Specifically, the downregulated genes were mainly related to regulation of lipid metabolic process, regulation of platelet cell cycle, ATPase activity, and nucleoside-triphosphatase activity (Figure 4(b)). Meanwhile, the upregulated genes were related to kinase activity, transcription factor activity, and response to reactive oxygen species (Figure 4(c)).

### 3.5. PPI Network and Subnetwork of DEGs

**Figure 5:**
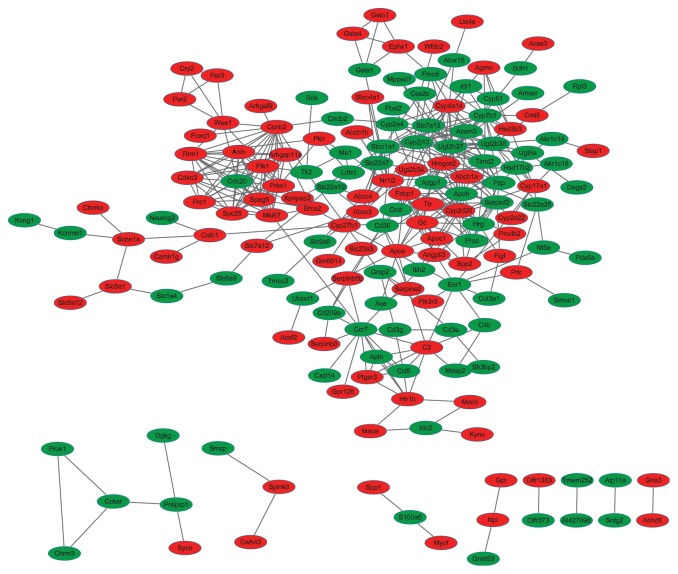
Interactome of the 355 genes showing 166 nodes and 411 edges in the protein-protein interaction map encompassing 4 clusters in DN. Genes are denoted as nodes in the graph, and interactions between them are presented as edges. Green indicates downregulated genes, whereas red indicates upregulated genes.

According to the PPI dataset downloaded from STRING, the PPI network consisted of 166 gene signatures and 411 interactions based on 355 DEGs. The network was binary, and all interactions were unweighted and undirected. The giant component, which included the majority of the entire network genes containing 143 nodes and 395 edges, was constructed (Figure 5). The top 10 genes in degree and betweenness centrality (BC) are listed in Table 4.

### 3.6. Confirmation with qPCR

**Figure 6:**
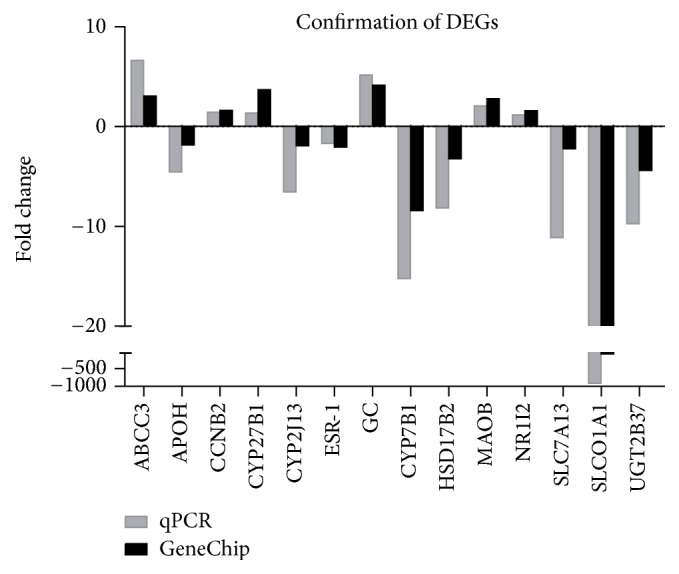
Confirmation of 14 representative DEGs by qPCR. The alterations in these genes at the mRNA level are similar between qPCR and GeneChip.

To validate the microarray results, we analyzed the mRNA levels of representative genes by using qPCR. As shown in Figure 6, the relative mRNA levels of* Abcc3, Ccnb2, Cyp27b1, Gc, Maob*, and* Nr1i2* in db/db mice were significantly higher, whereas the mRNA levels of* Apoh, Cyp2j13, Esr-1, Cyp7b1, Hsd17b2, Slc7a13, Slco1a1*, and* Ugt2b37* were lower compared with those in db/m mice (all *p* < 0.05). These outcomes were consistent with the microarray results.

## 4. Discussion

DN has been a widely known issue in public health because of the lack of available curative methods. The pathogenesis of DN remains highly complex to be fully understood. In this study, cDNA microarray analysis was employed to clarify the underlying mechanism in db/db mice, an ideal animal model for type 2 DN. A total of 164 downregulated genes and 191 upregulated genes were identified. Enriched significant GO terms and pathways based on these DEGs were also determined. In addition, 10 hub genes were selected from the main PPI network constructed from DEGs according to their BC and degree.

In this study, biological oxidation was determined to be the most significant pathway, which includes the upregulation of* Aoc1, BC021614, Cyp17a1, Cyp27b1, Cyp2d22, Cyp2d26, Cyp4a14, Ephx1, Gm10639, Gm3776, Gsto1, Maoa,* and* Maob* and downregulation of* Ces2c, Cndp2, Cyp2a4, Cyp51, *and* Cyp7b1*. Meanwhile,* Cyp17a1, Cyp27b1, Cyp2d22, Cyp2d26, Cyp4a14 Cyp2a4, Cyp51,* and* Cyp7b1* are members of the CYP450 superfamily, which is highly implicated in sustaining the redox balance and limiting the source of oxidative stress [9, 10]. Park et al. reported abnormal expression of multiple CYP450 isoforms in Zucker diabetic rats, indicating that high glucose maybe result to the disorder of CYP450 families [11]. Both monoamine oxidases A and B are flavin adenine dinucleotide-dependent enzymes. Deamination of noradrenaline, serotonin, and dopamine leads to the production of hydrogen peroxide (H2O2). MAO activity as a source of reactive oxygen (ROS) has recently been investigated [12], and hyperglycemia led to increased MAO-A expression and overproduction of ROS in diabetic rats [13]. Thus, the DEGs involved in biological oxidation may be participants in the pathogenesis of DN via oxidative stress.

An increasing number of studies have recently indicated that hormonal imbalance participated in the development of DN [14–16]. In the present study, steroid hormone analyses from the KEGG database were found to be significant in the enrichment analysis, which involves the upregulation of* Cyp17a1, Cyp2d22, Cyp2d26*, and* Ugt2b34* and downregulation of* Akr1c18, Cyp7b1, Hsd17b2, Ugt2b37,* and* Ugt2b38. HSD17B2* converts testosterone, dihydrotestosterone (DHT), and estradiol into their cognate inactive metabolites, A-dione, 5*α*A-dione, and estrone, respectively.* Cyp2d22* and* Cyp2d26*, a homolog of human* Cyp2d6*, exhibited a modest catalyzed activity from estradiol to 2-OH-E2 and 2-OH-E1 [17].* Cyp7b1* metabolizes 5*α*-androstane-3*β*,17*β*-diol (3*β*-Adiol) and dehydroepiandrosterone.* Ugt2b15*, an ortholog of* Ugt2b38*, catalyzes the conjugation of the 17*β*-hydroxy position of DHT, testosterone, and 3*α*-Diol [18]. Dysregulation of these genes led to reduced estrogen and elevated androgen levels. The close association between plasma leptin concentration and sex hormones has been demonstrated by Thomas et al. [19], while excess of circulating leptin may be an important contributor to the development of reduced androgens in male obesity [20]. These may partly account for the disorder of sex hormones regulated genes in leptin signaling deficient db/db mice. Estradiol administration to db/db mice improved DN [21]. Consistent with this finding,* Esr-1* encoded a receptor of estradiol, which is involved in DN [22], that was also significantly reduced in the current study.

We found that bile acid synthesis disorder may be associated with the pathogenesis of DN. This disorder includes bile acid and bile salt metabolism based on the reactome pathway, bile secretion from the KEGG pathway, and bile acid metabolic process from GO terms.* Abcc3* encodes MRP3, and* Abcc4* encodes MRP4. Both transport the taurine and glycine conjugates of bile acids, as well as the unconjugated bile acid cholate, into blood.* Slco1a1* encodes OATP1A1, which can transport unconjugated and conjugated bile acids into cells [23]. Decreased* Slco1a1* and increased* Abcc3* and* Abcc4* that were found in the present study would lead to loss of conjugated and unconjugated bile acid and bile salts in the cell. This finding is consistent with those in a previous study [24]. Cholic acid has been reported to alleviate kidney damage in db/db mice via modulation of renal lipid metabolism and modulation of fibrosis and inflammation [25]. The study from Liang and Tall suggested that supplement with leptin reversed the deficiency in bile acid synthesis and transport as well as lipoprotein and energy metabolism in ob/ob mice, indicating the important role of leptin in regulating bile acid metabolism [26]. Elevated bile acid pool in STZ-induced diabetic mice and spontaneous diabetic ob/ob mice also revealed that high glucose is an important regulator of bile acid synthesis [27]. FXR is a major receptor of bile acid. Its deficiency-exacerbated diabetic nephropathy occurred in type 1 diabetic rats [28]. In addition, OATP1A2 can also transport dehydroepiandrosterone sulfate, a precursor for the synthesis of steroid hormones [29]. The serum level of dehydroepiandrosterone sulfate was negatively correlated with severe diabetic nephropathy [30].

In addition to identifying the pathway involved in DN, we also distinguished 10 hub genes based on the PPI network from DEGs, some of which were not reported in DN. Notably,* Nr1i2* encodes the pregnane X receptor, a nuclear receptor. However, no direct evidence of PXR involvement in DN is available. On the one hand, PXR may play a more important role in regulating the metabolism of drugs being used to treat DN because of its general role in controlling drug responses [31]. On the other hand, PXR influences macrophage sensitivity to cholesterol. Abnormal lipid metabolism in the kidney has been implicated in the development of DN. The current study found elevated PXR in db/db mice, suggesting that PXR regulates lipid metabolism and drug response in DN.

Cyclin B2 encoded by* Ccnb2* is a member of the cyclin family, specifically the B-type cyclins. Cyclin B2 is associated with p34cdc2 and is an essential component of the cell cycle regulatory machinery. Cyclin B2 is primarily associated with the Golgi region. It also binds to transforming growth factor beta R2. Thus, cyclin B2/cdc2 may play a key role in transforming growth factor beta-mediated cell cycle control.* Ccnb2* increased in podocytes in rats with experimental membranous nephropathy. Increased* Ccnb2* expression promotes the proliferation of the cell, which may be associated with the proliferation of mesangial and tubular cells in high glucose, along with elevated TGF-*β*1 expression. In the present study, db/db mice exhibited upregulation of* Ccnb2* at the mRNA level, suggesting its contribution to DN via interference with cell G2/M phage.

*Cyp2j13* is an ortholog of* Cyp2j2* in humans, which is mainly expressed in the kidneys of mice.* Cyp2j13* is responsible for the conversion of arachidonic acid to EETs via the epoxygenase pathway [32]. Several reports suggested that EET production was significantly decreased in the kidney of STZ-induced diabetic rats and high glucose-treated proximal tubular epithelial cells [33–35]. Inhibition of EETs results in overproduction, cellular hypertrophy, and accumulation of fibronectin and collagen IV in high glucose incubated tubular cells [34]. Furthermore, overexpression of endothelial* Cyp2j2* also attenuated renal injuries in diabetic rats [36].* Cyp2j13* was reduced in the study, suggesting its involvement in DN by decreasing EET production.

*Cyp27b1* encodes a member of the cytochrome P450 superfamily of enzymes, the enzyme that catalyzes the conversion of 25-hydroxyvitamin D3 (25(OH)D) to 1-alpha,25-dihydroxyvitamin D3 (1,25(OH)2D), the active form of vitamin D3. It then binds to the vitamin D receptor and regulates calcium metabolism. Thus, this enzyme plays an important role in normal bone growth, calcium metabolism, and tissue differentiation via regulation of biologically active vitamin D.* Cyp27b1* mRNA expression, which was increased in the kidney from diabetic DBA mice, revealed dysregulation of the vitamin D endocytic pathway [37, 38]. The current study supports that* Cyp27b1* could be participants in the development of DN via regulation of vitamin D signaling.

*Apoh* encodes *β*2GPI, a 54.2 kDa single-chain protein containing four N-linked glycosylation sites, which is essential for binding phospholipids.* Apoh* also binds to various kinds of negatively charged substances such as heparin, phospholipids, and dextran sulfate. It plays multiple roles in coagulation and fibrinolytic pathways, placental homeostasis, athermanous plaque formation, endothelial cell activation, and apoptotic mechanisms [39]. The transcriptome analysis from human DN revealed the downregulation of* Apoh* in the diabetic tubule [5]. Increased urinary *β*2GPI excretion was proven to be a biomarker of impaired tubular reabsorption of protein in diabetics without clinical proteinuria [40]. Contradictory to these findings, reduced *β*2GPI ameliorated renal morphometric damage and kidney function in STZ-induced diabetic rats via inhibition of the TGF-*β*1-p38 MAPK pathway [41]. The present study revealed reduced expression of* Apoh*. However, the role of* Apoh* in DN has yet to be elucidated.

## 5. Conclusion

The current findings together indicate that upregulation of 164 probesets with genes and downregulation of 191 probesets. The DEGs were enriched for biological oxidation, steroid hormone biosynthesis, and bile acid metabolism. Some recognized hub genes, such as* Ccnb2*,* Nr1i2* and others, provide new insights into the molecular pathogenesis of DN.

## Figures and Tables

**Table 1:** Primers used in qPCR.

	Forward	Reverse
*Abcc3*	CCATTGACTTGGAGACGGATG	CGCAATGAGGTTGACTGGAG
*Apoh*	TGCATGGCGACAAAATTCAC	CCGTTTTCCAGAAAGCCAGAG
*Ccnb2*	TTGCCTGTCTCAGAAGGTGC	GGGGAGGCCAGGTCTTTGATG
*Cyp2j13*	CCACCCCAGACATCTTCAAT	AATTGTTCTCCGAGGCAAGCT
*Cyp27b1*	ACCCATTTGCATCTCTTCCCTT	CGGGTCATGGGCTTGATAGG
*Esr1*	CGTTTCTGTCCAGCACCTTG	CATGTGCCGGATATGGGAAAG
*Gc*	GGACAAAAACACCCAACACCT	CCATCTCTGTGGTGCTTGATT
*Maob*	ACTGAAACAGCCTCACACTGG	GGTACTGGTAATGGGTCGTGC
*Nr1i2*	TCGAAGACCCTAATGGTGGC	GAGCAGGATATGGCCGACTAC
*Slc7a13*	TGTTTTGTGCCCTGAATGTCC	CCCAACGCTATGAATGTGAAC
*Slco1a1*	TTCCGGCACCTGTTTACTTTG	TAGAATGAAGACTGCGGGGAG
*Ugt2b37*	TCCTTTGTTTGGAGAACAGCAT	AGGCTGGTCATGGTGAATGG
*Hsd17b2*	AATCATCAGACAGGAGCTTGAC	CCTCTCTTTCAAGGTCGGGAT
Cyp7b1	GTATTATATTCTTCGGCATCCTG	CATATCCTCCTGCACTTCTCG
*β-actin*	ACCCTAAGGCCAACCGTGAAAAG	CATGAGGTAGTCTGTCAGGT

**Table 2:** Top 10 enriched GO terms for DEGs sorted by *p* value (ascending).

GOID	GO term	Term *p* value corrected with Bonferroni step down	Genes cluster #1	Genes cluster #2
GO:0030193	Regulation of blood coagulation	7.10*E* − 07	*[Apoe, Ptger3, Serpine2]*	*[Apoh, Cd209a, Cd36, Hrg, Proc, Rpl3, Serpinf2, Tc2n]*
GO:0015291	Secondary active transmembrane transporter activity	1.00*E* − 06	*[Gm6614, Slc22a29, Slc5a1, Slc6a12, Slc7a12, Slc8a1, Slco4a1]*	*[Mfsd2a, Slc1a4, Slc22a19, Slc22a26, Slc22a28, Slc6a9, Slc7a13, Slc9a8, Slco1a1, Tmco3]*
GO:0008514	Organic anion transmembrane transporter activity	3.30*E* − 06	*[Abcc4, Gm6614, Slc22a29, Slc6a12, Slc7a12, Slco4a1]*	*[Slc1a4, Slc22a19, Slc22a26, Slc22a28, Slc22a7, Slc6a9, Slc7a13, Slco1a1]*
GO:0019216	Regulation of lipid metabolic process	1.90*E* − 05	*[Abhd6, Angptl3, Angptl8, Apoc1, Apoe, C3, Cyp17a1, Cyp27b1, Fabp1, Pdk1, Pik3r3, Prox1]*	*[Akr1c18, Apoh, Esr1, Kcnma1, Nt5e]*
GO:0004497	Monooxygenase activity	3.10*E* − 05	*[Agmo, Cyp17a1, Cyp27b1, Cyp2d22, Cyp2d26, Cyp4a14]*	*[Akr1c18, Cyp2a4, Cyp2j13, Cyp51, Cyp7b1, Fmo5]*
GO:0015297	Antiporter activity	1.00*E* − 04	*[Slc22a29, Slc7a12, Slc8a1]*	*[Slc22a19, Slc22a26, Slc22a28, Slc7a13, Slc9a8, Tmco3]*
GO:0009636	Response to toxic substance	1.40*E* − 04	*[Aoc1, Apoe, Cyp17a1, Ephx1, Fabp1, Gria3, Gsta4, Gsto1, Ltc4s, Maob, Nupr1]*	*[Bdh1, Ccl5, Cd36, Ubiad1]*
GO:0015908	Fatty acid transport	1.80*E* − 03	*[Abcc4, Apoe, Fabp1]*	*[Ace, Cd36, Crot, Mfsd2a]*
GO:0016765	Transferase activity, transferring alkyl or aryl (other than methyl) groups	1.80*E* − 03	*[BC021614, Gm10639, Gsta4, Gsto1, Ltc4s]*	*[Agps, Ubiad1]*
GO:0022600	Digestive system process	1.90*E* − 03	*[Cyp27b1, Fabp1, Ptger3, Slc5a1]*	*[Cckar, Cd36, Chrm3, Kcnma1]*

**Table 3:** Top 10 enriched pathway terms of DEGs sorted by *p* value (ascending).

GO term	Ontology source	Term *p* value corrected with Bonferroni step down	Genes cluster #1	Genes cluster #2
Biological oxidations	REACTOME_10.02.2016	520.0*E* − 9	*[Aoc1, BC021614, Cyp17a1, Cyp27b1, Cyp2d22, Cyp2d26, Cyp4a14, Ephx1, Gm10639, Gm3776, Gsto1, Maoa, Maob]*	*[Ces2c, Cndp2, Cyp2a4, Cyp51, Cyp7b1]*
Bile acid and bile salt metabolism	REACTOME_10.02.2016	120.0*E* − 6	*[Abcc3, Gm6614]*	*[Akr1c14, Akr1c18, Amacr, Cyp7b1, Slco1a1]*
Steroid hormone biosynthesis	KEGG_10.02.2016	440.0*E* − 6	*[Cyp17a1, Cyp2d22, Cyp2d26, Ugt2b34]*	*[Akr1c18, Cyp7b1, Hsd17b2, Ugt2b37, Ugt2b38]*
Bile secretion	KEGG_10.02.2016	680.0*E* − 6	*[Abcb1a, Abcb1b, Abcc3, Abcc4, Ephx1, Slc5a1]*	*[Slc22a7, Slco1a1]*
Metabolism of fat-soluble vitamins	REACTOME_10.02.2016	770.0*E* − 6	*[Apoe, Cyp27b1, Gc, Ttr]*	*[Akr1c14, Akr1c18, Ubiad1]*
Metapathway biotransformation	WikiPathways_10.02.2016	3.5*E* − 3	*[Cyp17a1, Cyp27b1, Ephx1, Gm3776, Gsta4, Gsto1]*	*[Chst11, Cyp51, Cyp7b1, Fmo5]*
Glutathione conjugation	REACTOME_10.02.2016	6.2*E* − 3	*[BC021614, Gm10639, Gm3776, Gsto1]*	*[Cndp2]*
Generation of second messenger molecules	REACTOME_10.02.2016	93.0*E* − 3		*[Cd3e, Cd3g, Grap2]*
Peroxisomal lipid metabolism	REACTOME_10.02.2016	93.0*E* − 3		*[Agps, Amacr, Crot]*
Cyclin A/B1 associated events during G2/M transition	REACTOME_10.02.2016	93.0*E* − 3	*[Ccnb2, Plk1, Wee1]*	

**Table 4:** Top 10 hub genes from PPI network.

Name	Betweenness centrality	Degree	Fold change
*Nr1i2*	0.245983	14	−1.58
*Esr1*	0.22107	9	−1.71
*Ccr7*	0.127205	13	−1.59
*C3*	0.085003	9	−1.58
*Slc7a13*	0.082573	14	−2.23
*Slco1a1*	0.080427	16	−84.31
*Apoh*	0.078909	17	−1.84
*Ccnb2*	0.07628	15	1.6
*Cyp2j13*	0.067509	17	−1.94
*Fabp1*	0.066322	12	1.62
*Ugt2b37*	0.065938	20	−4.4

## References

[1] Nasri H. (2013). On the occasion of the world diabetes day 2013; Diabetes education and prevention; a nephrology point of view. *Journal of Renal Injury Prevention*.

[2] Ghaderian S. B., Hayati F., Shayanpour S., Beladi Mousavi S. S. (2015). Diabetes and end-stage renal disease; a review article on new concepts. *Journal of Renal Injury Prevention*.

[3] Pourghasem M., Shafi H., Babazadeh Z. (2015). Histological changes of kidney in diabetic nephropathy. *Caspian Journal of Internal Medicine*.

[4] Tang S. C., Chan G. C., Lai K. N. (2016). Recent advances in managing and understanding diabetic nephropathy. *F1000Research*.

[5] Woroniecka K. I., Park A. S. D., Mohtat D., Thomas D. B., Pullman J. M., Susztak K. (2011). Transcriptome analysis of human diabetic kidney disease. *Diabetes*.

[6] Schmid H., Boucherot A., Yasuda Y. (2006). Modular activation of nuclear factor-*κ*B transcriptional programs in human diabetic nephropathy. *Diabetes*.

[7] Watanabe T., Tomioka N. H., Doshi M., Watanabe S., Tsuchiya M., Hosoyamada M. (2013). Macrophage migration inhibitory factor is a possible candidate for the induction of microalbuminuria in diabetic db/db mice. *Biological & Pharmaceutical Bulletin*.

[8] Tesch G. H., Lim A. K. H. (2011). Recent insights into diabetic renal injury from the db/db mouse model of type 2 diabetic nephropathy. *American Journal of Physiology—Renal Physiology*.

[9] Bhattacharyya S., Sinha K., Sil P. C. (2014). Cytochrome P450s: mechanisms and biological implications in drug metabolism and its interaction with oxidative stress. *Current Drug Metabolism*.

[10] Hrycay E. G., Bandiera S. M. (2015). Involvement of cytochrome P450 in reactive oxygen species formation and cancer. *Advances in Pharmacology*.

[11] Park S. Y., Kim C. H., Lee J. Y. (2016). Hepatic expression of cytochrome P450 in Zucker diabetic fatty rats. *Food and Chemical Toxicology*.

[12] Manni M. E., Bigagli E., Lodovici M., Zazzeri M., Raimondi L. (2012). The protective effect of losartan in the nephropathy of the diabetic rat includes the control of monoamine oxidase type A activity. *Pharmacological Research*.

[13] Umbarkar P., Singh S., Arkat S., Bodhankar S. L., Lohidasan S., Sitasawad S. L. (2015). Monoamine oxidase-A is an important source of oxidative stress and promotes cardiac dysfunction, apoptosis, and fibrosis in diabetic cardiomyopathy. *Free Radical Biology & Medicine*.

[14] Xu Q., Wells C. C., Garman J. H., Asico L., Escano C. S., Maric C. (2008). Imbalance in sex hormone levels exacerbates diabetic renal disease. *Hypertension*.

[15] Costacou T., Fried L., Ellis D., Orchard T. J. (2011). Sex differences in the development of kidney disease in individuals with type 1 diabetes mellitus: a contemporary analysis. *American Journal of Kidney Diseases*.

[16] Maric C. (2009). Sex, diabetes and the kidney. *American Journal of Physiology—Renal Physiology*.

[17] Lee A. J., Cai M. X., Thomas P. E., Conney A. H., Zhu B. T. (2003). Characterization of the oxidative metabolites of 17*β*-estradiol and estrone formed by 15 selectively expressed human cytochrome P450 isoforms. *Endocrinology*.

[18] Turgeon D., Carrier J.-S., Lévesque E., Hum D. W., Bélanger A. (2001). Relative enzymatic activity, protein stability, and tissue distribution of human steroid-metabolizing UGT2B subfamily members. *Endocrinology*.

[19] Thomas T., Burguera B., Melton L. J. (2000). Relationship of serum leptin levels with body composition and sex steroid and insulin levels in men and women. *Metabolism: Clinical and Experimental*.

[20] Isidori A. M., Caprio M., Strollo F. (1999). Leptin and androgens in male obesity: evidence for leptin contribution to reduced androgen levels. *The Journal of Clinical Endocrinology and Metabolism*.

[21] Chin M., Isono M., Isshiki K. (2005). Estrogen and raloxifene, a selective estrogen receptor modulator, ameliorate renal damage in db/db mice. *American Journal of Pathology*.

[22] Inada A., Inada O., Fujii N. L. (2016). Adjusting the 17*β*-estradiol-to-androgen ratio ameliorates diabetic nephropathy. *Journal of the American Society of Nephrology*.

[23] Kullak-Ublick G. A., Hagenbuch B., Stieger B. (1995). Molecular and functional characterization of an organic anion transporting polypeptide cloned from human liver. *Gastroenterology*.

[24] More V. R., Wen X., Thomas P. E., Aleksunes L. M., Slitt A. L. (2012). Severe diabetes and leptin resistance cause differential hepatic and renal transporter expression in mice. *Comparative Hepatology*.

[25] Jiang T., Wang X. X., Scherzer P. (2007). Farnesoid X receptor modulates renal lipid metabolism, fibrosis, and diabetic nephropathy. *Diabetes*.

[26] Liang C.-P., Tall A. R. (2001). transcriptional profiling reveals global defects in energy metabolism, lipoprotein, and bile acid synthesis and transport with reversal by leptin treatment in ob/ob mouse liver. *Journal of Biological Chemistry*.

[27] Li T., Francl J. M., Boehme S. (2012). Glucose and insulin induction of bile acid synthesis: mechanisms and implication in diabetes and obesity. *The Journal of Biological Chemistry*.

[28] Wang X. X., Jiang T., Shen Y. (2010). Diabetic nephropathy is accelerated by farnesoid X receptor deficiency and inhibited by farnesoid X receptor activation in a type 1 diabetes model. *Diabetes*.

[29] Kullak-Ublick G.-A., Fisch T., Oswald M. (1998). Dehydroepiandrosterone sulfate (DHEAS): identification of a carrier protein in human liver and brain. *FEBS Letters*.

[30] Kanauchi M., Nakajima M., Dohi K. (2001). Dehydroepiandrosterone sulfate and estradiol in men with diabetic nephropathy [2]. *Nephron*.

[31] Zhou C., King N., Chen K. Y., Breslow J. L. (2009). Activation of PXR induces hypercholesterolemia in wild-type and accelerates atherosclerosis in apoE deficient mice. *Journal of Lipid Research*.

[32] Wray J. A., Sugden M. C., Zeldin D. C. (2009). The epoxygenases CYP2J2 activates the nuclear receptor PPAR*α* in vitro and in vivo. *PLoS ONE*.

[33] Luo P., Zhou Y., Chang H.-H. (2009). Glomerular 20-HETE, EETs, and TGF-*β*1 in diabetic nephropathy. *American Journal of Physiology—Renal Physiology*.

[34] Eid S., Maalouf R., Jaffa A. A. (2013). 20-HETE and EETs in diabetic nephropathy: a novel mechanistic pathway. *PLoS ONE*.

[35] Shapiro H., Theilla M., Attal-Singer J., Singer P. (2011). Effects of polyunsaturated fatty acid consumption in diabetic nephropathy. *Nature Reviews Nephrology*.

[36] Chen G., Wang P., Zhao G. (2011). Cytochrome P450 epoxygenase CYP2J2 attenuates nephropathy in streptozotocin-induced diabetic mice. *Prostaglandins & Other Lipid Mediators*.

[37] Pei F., Li B.-Y., Zhang Z. (2014). Beneficial effects of phlorizin on diabetic nephropathy in diabetic db/db mice. *Journal of Diabetes and Its Complications*.

[38] Wang Y., Zhou J., Minto A. W. (2006). Altered vitamin D metabolism in type II diabetic mouse glomeruli may provide protection from diabetic nephropathy. *Kidney International*.

[39] Miyakis S., Giannakopoulos B., Krilis S. A. (2004). Beta 2 glycoprotein I-function in health and disease. *Thrombosis Research*.

[40] Lapsley M., Flynn F. V., Sansom P. A. (1993). *β*2-glycoprotein-1 (apolipoprotein H) excretion and renal tubular malfunction in diabetic patients without clinical proteinuria. *Journal of Clinical Pathology*.

[41] Wang T., Chen S.-S., Chen R., Yu D.-M., Yu P. (2015). Reduced beta 2 glycoprotein i improve diabetic nephropathy via inhibiting TGF-*β*1-p38 MAPK pathway. *International Journal of Clinical and Experimental Medicine*.

